# Neuritin Attenuates Neuronal Apoptosis Mediated by Endoplasmic Reticulum Stress In Vitro

**DOI:** 10.1007/s11064-018-2553-4

**Published:** 2018-05-22

**Authors:** Xiaokun Sun, Linzhi Dai, Hang Zhang, Xuejun He, Fandi Hou, Wengao He, Shijun Tang, Dong Zhao

**Affiliations:** 1 Department of Neurosurgery, First Affiliated Hospital of Shihezi University, Shihezi, 832000 China; 2 Department of Neurosurgery, The People’s Hospital of Xinjiang Uygur Autonomous Region, Urumchi, 830001 China

**Keywords:** Neuritin, Endoplasmic reticulum stress, Neurons, Apoptosis

## Abstract

Neuritin is an extracellular glycophosphatidylinositol-linked protein that promotes neuronal survival, differentiation, function, and repair, but the exact mechanism of this neuroprotective effect remains unclear. Meanwhile, endoplasmic reticulum stress (ERS) induced apoptosis is attracting increased attention. In this work, we hypothesized that neuritin inhibited ERS to protect cortical neurons. To check this hypothesis, we exposed primary cultured cortical neurons to oxygen and glucose deprivation (OGD) for 45 min followed by reperfusion (R) to activate ERS. We then performed resuscitation for 6, 12, 24, and 48 h. ERS-related factors such as glucose-regulated protein 78 (GRP78), caspase-12 and CHOP were detected by Western blotting and quantitative real-time polymerase chain reaction assay. Apoptosis was assessed by Annexin V binding and propidium iodide staining. Ultrastructural changes of endoplasmic reticulum were observed under a transmission electron microscope. Results showed that GRP78 expression significantly increased at 12, 24, and 48 h and peaked at 24 h. Caspase-12 and CHOP expression significantly increased in a time-dependent manner at 12, 24, and 48 h. GRP78, caspase-12 and CHOP expression as well as apoptosis rate of primary cultured neurons and the ultrastructural changes of endoplasmic reticulum in the OGD/R + neuritin group significantly improved compared with the OGD/R group. In conclusion, the neuroprotection function of neuritin may be involved in ERS pathways.

## Introduction

Brain ischemia hypoxia is due to decreased blood supply to the brain and can lead to significant neuronal apoptosis in the ischemic area [1]. In turn, neuronal apoptosis in functional brain areas can lead to long-term neurologic sequelae and even death, which translate to heavy economic and social burden [2]. Apoptosis pathways mainly include death-receptor activation (exogenous pathway), mitochondrial-damage pathway (endogenous pathway), and apoptosis pathway initiated by endoplasmic reticulum stress (ERS). The first two are classical apoptotic pathways, but the ERS pathway is a recently discovered apoptotic pathway [3]. ERS can reportedly be activated in neurons damaged by cerebral ischemia [4].

The endoplasmic reticulum (ER), known as the cell machinery, possesses a huge membrane [5]. It plays critical roles in wide ranging of processes, including protein synthesis, folding, modification and transport; phospholipid and steroid synthesis and distribution; calcium ion storage within its lumen; and regulation of calcium ion release into the cytoplasm [6, 7]. The biological function of ER enables it to be easily disturbed by physiological and pathological conditions, including glucose deprivation, viral infection, Ca^2+^ depletion of ER, and ischemia/reperfusion, which lead to the accumulation of misfolded and unfolded proteins [8]. Such accumulation of abnormal proteins, which harm cellular environmental homeostasis, is called ERS [7, 9]. Previous studies have indicated that ERS is involved in some neurological diseases, Such as hypoxic ischemic encephalopathy [1, 10], SAH [11, 12], neurodegeneration diseases including Alzheimer’s disease (AD), Parkinson’s disease (PD), amyotrophic lateral sclerosis (ALS), and prion-related diseases [6, 13]. However, an adaptive mechanism of cells, called the unfolded protein reaction (UPR) can relieve the stress [5, 14]. UPR signal pathways include the inositol-requiring enzyme 1 (IRE1) pathway, activating transcription factor 6 (ATF-6) pathway, and protein kinase R-like endoplasmic reticulum kinase (PERK) pathway. Cells can be protected from short and weak stress by these pathways. However, severe stress that exceed the self-protection ability of cells contribute to apoptosis [14, 15]. The 78-kDa glucose-regulated protein GRP78, also known as BiP and HSP5a, play an important role in UPR for restoring protein balance during ERS [13, 16, 17]. The C/EBP homologous protein (CHOP/GADDDD153) and the caspase-12 dependent pathway are associated with ERS-induced apoptosis [17–19].

Neuritin, also identified as the candidate plasticity gene 15 (*cpg15*), is a neurotrophic-related neurotrophic factor, first acknowledged and characterized upon screening for genes regulated in the rat hippocampal dentate gyrus by kainite-induced seizures [20], *cpg15* is highly expressed in the nervous system [21, 22], especially in sensory neurons [23], hippocampus, visual cortex, and external granular layer of the cerebellum [24]. Neuritin can also promote synaptic growth, axonal regeneration, and nerve cell maturation [6, 23]; protect motor neurons and retinal ganglion cells [25]; and prevent nerve cells from apoptosis [26]. Neuritin also reportedlly plays neuroprotective roles in spinal cord injury, subarachnoid hemorrhage (SAH) [11], chronic unpredictable stress (CUS) [27], diabetic neuropathy [23], and Alzheimer’s disease [24]. In our previous study, we found that neuritin has a neuroprotective effect on SAH in vivo [11]. Our finding suggests that neuritin attenuates early brain injury (EBI) after SAH by improving the clinical scale of brain edema and lowering the neural-cell apoptosis [11]. These reports indicate that neuritin may have a therapeutic effect on some central nervous system diseases, but its mechanism remains unclear.

In this study, we used an in vitro model that was established on primary cultured neurons suffering from OGD/R to explore the anti-apoptosis effect of neuritin in ERS. We detected the production of GRP78 which plays important roles in UPR, CHOP, and caspase-12 that are related to ERS-induced apoptosis. Changes in rough ER ultrastructures were observed as well.

## Materials

### Ethics Statement

This study was approved by the Institutional Animal Care and Use Committee of the First Affiliated Hospital of Medical College, Shihezi University. All efforts were made to reduce the number and minimize the pain and suffering of animals.

### Animal

Sprague-Dawley (SD) rats born within 24 h and weighing (7 ± 0.5) g were purchased from the Experimental Animal Center of Xinjiang Uygur Autonomous Region, China. Three adult rats (male:female = 1:2) were housed per cage and under a reversed 12 h light/dark cycle with ad libitum food and water. Pregnant rats are kept separately in a quiet environment.

### Reagents

DMEM-low glucose, DMEM-high glucose, B27 supplement, and Neurobasal-A medium (1×) were purchased from Gibco/Life Technologies (Grand Island, NY, USA). Trypsin (0.25%) and Penicillin/Streptomycin (10,000 U/mL) were purchased from HyClong (Logan, USA). PBS (20×) and poly-l-lysine were purchased from Shanghai Bio engineering Co., Ltd.(Shanghai, China). Fetal bovine serum (FBS; 1×) was purchased from Zhejiang Tianhang Biological Polytron Technologies Inc (Zhejiang, China). Neuritin was purchased from PEPROTECH (Princeton, USA).

### Primary Culture of Cortical Neurons

SD rats born within 24 h were anesthetized by placing in a refrigerator at − 20 °C for 15 min. Neonatal rats were rinsed twice with 75% ethanol for 1 min, to disinfect the skin. The rats were beheaded, and their scalps, meninges, and skulls were removed to expose the brain cortex. The cerebral cortex was removed to the extraction medium (DMEM-high glucose with 100 U/mL penicillin/streptomycin) and placed on ice. Small pieces of the cerebral cortex were digested using 0.25% trypsin for 15–20 min at 37 °C. DMEM-high glucose with 10% FBS was used to terminate digestion. Cortical neurons were dissociated after suitable mechanical percussion. Cortical neurons were made up to cell suspension using DMEM-high glucose with 10% FBS after filtering through 200 mesh and centrifuged at 1000 r/min for 5 min.

The cell suspension was cultured in six-well plates pretreated with poly-l-lysine after being counted under a phase contrast microscope to ensure that the cell density was greater than 1 × 10^6^/mL in inoculation medium (DMEM-high glucose with 10% fetal bovine serum and 100 U/mL penicillin/streptomycin) for 3–4 h at 37 °C, thereby allowing cortical neurons to adhere and survive. The inoculation medium was replaced with growth medium after 3–4 h (Neurobasal-A medium (1×) with 2% B27 and 100 U/mL penicillin/streptomycin). Half of the growth medium was replaced every 2 days, until the cortical neurons matured after culturing for 7–8 days in a cell-culture incubator with 95% air and 5% CO_2_.

### Establishment of Reperfusion (R) Model After Oxygen–Glucose Deprivation (OGD) and Experimental Groups

Old growth medium was fully replaced with DMEM-low glucose medium when cortical neurons were cultured for 7–8 days. Subsequently, the six-well plates were placed in a hypoxia incubator filled with 95% N_2_ and 5% CO_2_ at 37 °C for 45 min. The DMEM-low glucose medium was then replaced. The oxygen–glucose deprivation/reperfusion (OGD/R) group (*n* = 32) was replaced with the growth medium, and the OGD/R + neuritin group (*n* = 32) was replaced with growth medium and neuritin (200 ng/mL). The control group (*n* = 32) was cultured on the same plates, and the growth medium was placed punctually and kept in the same incubator without OGD/R. The non-OGD/R + neuritin group (*n* = 32) was added with neuritin (200 ng/mL) and not subjected to OGD/R. Cortical neurons were harvested for further experiments after culturing again in a cell culture incubator with 95% air and 5% CO_2_ for 6, 12, 24, and 48 h. Eight SD rats born within 24 h per group were used for Western blotting, Quantitative real-time polymerase chain reaction (qRT-PCR), flow cytometry, and transmission electron microscopy.

### Western Blotting

Western blotting was performed to detect the expression of GRP78, caspase-12, and CHOP. The total protein of each group was extracted afterdissociation in a lysis buffer (1 mL of RIPA, 10 µL of PMSF; 30 min on ice) and centrifuged at 12,000 rpm (10 min at 4 °C). Total proteins were quantified by the BCA method, evaluated by SDS-PAGE electrophoresis and transferred to SDS-PAGE membranes. The membranes were blocked with 5% milk and Tris-Buffered Saline and washed by 1× TBST buffer. Rabbit anti-rat polyclonal anti-GRP78 (1:500; ab21685, Abcam, Cambridge, MA), anti-caspase-12 (1:1000; ab62484, Abcam) and anti-CHOP (1:500; ab11419, Abcam) were used as primary antibodies. The blots were incubated at room temperature for 3 h. Then, a horseradish peroxidase-conjugated rabbit anti-goat antibody was used as a secondary antibody (1:10,000 dilution, Zhongshan Jinqiao Biotechnology Co., Beijing, China) in 5% non-fat milk in TBST for 1 h at room temperature. Protein bands were detected by an enhanced chemiluminescence system. Quantity One software was applied to analyze the bands.

### qRT-PCR Assay

Total RNA was extracted from the cultured neurons using Trizol reagents (Sangon Biotech, Shanghai, China), and 500 ng of RNA in each group was reverse transcribed into cDNA using AMV First-Strand cDNA Synthesis Kit (Sangon Biotech, Shanghai, China) following the manufacturer’s protocol. qRT-PCR was operated on a LightCycler480 Software Setup (Applied Biosystems, Irvine, CA, USA) with Power SYBR Green (Applied Biosystems, Irvine, CA, USA). The PCR mixtures were pre-heated at 95 °C for 3 min and then at 95 °C for 3 min to activate Ampli Taq Gold DNA polymerase. Subsequently, all reactions were subjected to 40 cycles of amplification (denaturation at 95 °C for 15 s and annealing/extension at 60 °C for 40 s). β-actin was used as a reference gene. The expression of each targeted gene was analyzed using the 2^−[ΔΔCt]^ method, ∆∆Ct = (Ct_target mRNA_ − Ct_β−actin_)_other groups_ − (Ct_target mRNA_ − Ct_β−actin_)_control group_. Three independent experiments were carried out. The primer sequences for GRP78, caspase-12, CHOP, and β-actin were:

- GRP78: 118GAACCAACTCACGTCCAACC(F)
- GRP78: 118AACCACCTTGAATGGCAAGA(R)
- Caspase-12: 116GGACATGCTGGATGGAGTTT(F)
- Caspase-12: 116CCAGGTTCTCAGCTTTGCTC(R)
- CHOP: 127CTGGAAGCCTGGTATGAGGA(F)
- CHOP: 127GGGATGCAGGGTCAAGAGTA(R)
- β-actin: 137CAACCTTCTTGCAGCTCCTC(F)
- β-actin: 137CGGTGTCCCTTCTGAGTGTT(R)

### Flow Cytometry with ANNEXIN V-FITC/PI Staining

Flow cytometry was performed as previously described to quantitatively evaluate neuronal apoptosis [17]. More than 1 × 10^5^ cultured cortical neurons were collected, washed three times with PBS, and finally digested with 0.25% trypsin without EDTA (1 mL; 5 min). The digested neurons were centrifuged at 2000 rpm (10 min at room temperature). The cortical neurons were resuspended in binding buffer (500 µL). Annexin V-FITC (10 µL) and PI (5 µL) (MultiSciences, Hangzhou, China) were sequentially added and mixed with cortical neurons. The mixture was incubated at room temperature away from light for 5–15 min. Cells in each group were tested and promptly analyzed by flow cytometry (FACSAria III, Beckman-Coulter, USA). The experiment was repeated three times.

### Ultrastructures of Neurons

The cultured neurons in the control group, non-OGD/R group, OGD/R group, and neuritin group, were fixed in 2.5% glutaraldehyde, dyed with osmic acid, dehydrated with acetone in gradient concentration, and embedded in epoxy resin. These neurons were observed using a transmission electron microscopy (JEM-1230, JEOL, Japan). Changes in ultrastructures of neurons in the ER were observed under 80 kV.

### Statistic Analysis

Values are expressed as the mean ± SD of at least three independent experiments. Statistical analyses were performed by SPSS 18.0. Differences among all groups were analyzed by one-way ANOVA and multiple comparisons were conducted with the post-hoc Bonferroni test. Significances was accepted at *p* < 0.05.

## Results

### OGD/R Increased GRP78 Expression in Cultured Neurons

OGD is believed to be one of the main causes of ERS, and can thus be used to estiblish an ERS model [14]. GRP78, as a vital regulator of ER function, plays a crucial role in ERS [28]. The onsets of UPR and ERS reportedlly increases GRP78 expression. Accordingly, Western blotting analysis and qRT-PCR were used to study the expression of GRP78 protein and mRNA to determine whether OGD/R induced UPR and ERS.

**Fig. 1:**
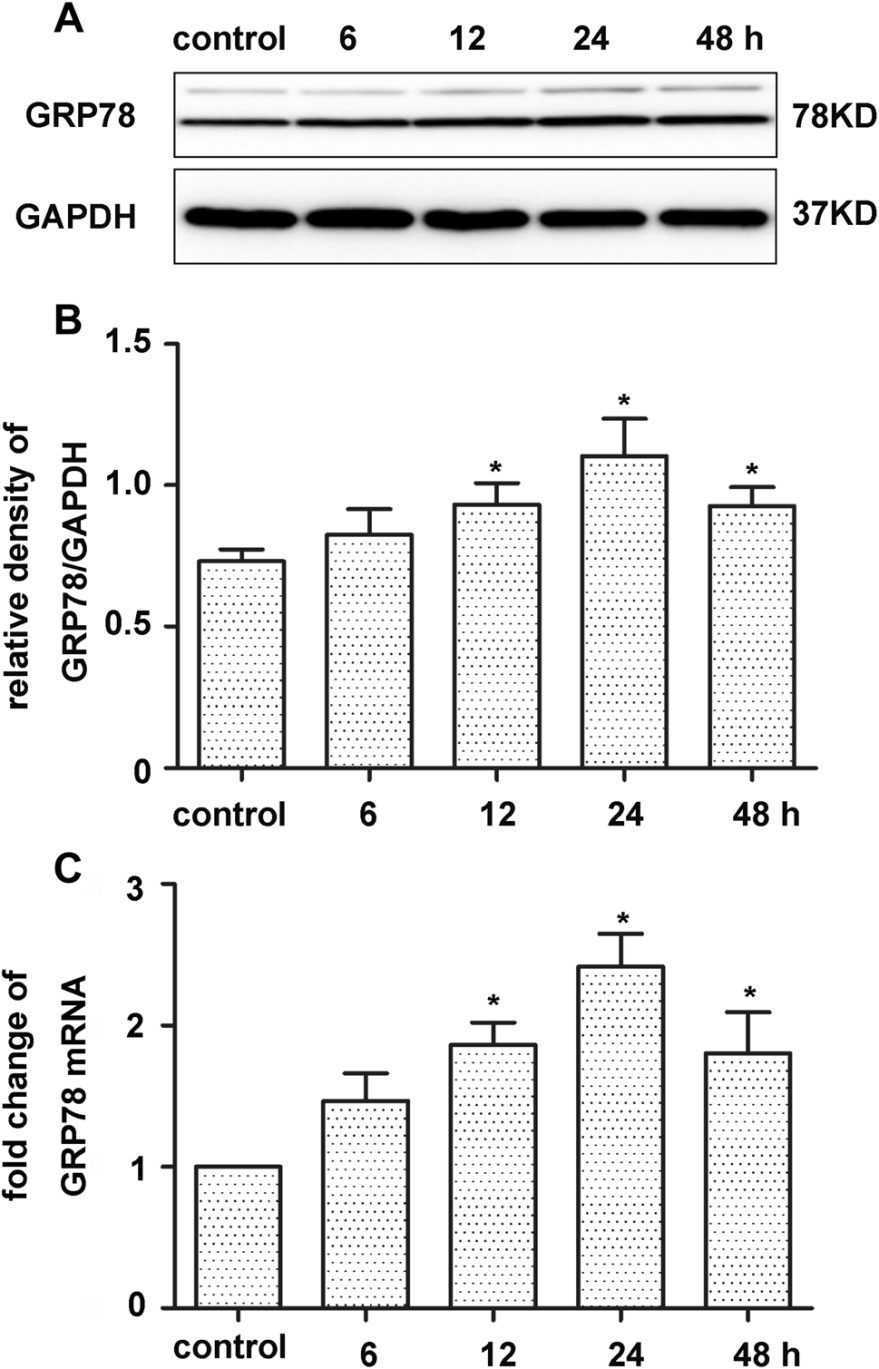
Effect of OGD/R on GRP78 expression in primary cultured neurons. **a, b** Protein expression of GRP78 in the OGD/R group. GAPDH was used for loading control and band-density normalization. Results represent the mean ± SD. *p < 0.05 versus the control group. The experiment was repeated six times. **c** mRNA levels of GRP78 in the OGD/R group Results represent the mean ± SD. *p < 0.05 versus the control group. The experiment was repeated three times

As shown in Fig. 1, compared with the control group, the expression of GRP78 was upregulated significantly after 12, 24, and 48 h in OGD/R group (*p* < 0.05) as detected by Western blotting and qRT-PCR assay. GRP78 expression peaked at 24 h. These results demonstrated that OGD/R resuledt in the ERS of cultured neurons..

### OGD/R Increased the Expression of Caspase-12 and CHOP in Cultured Neurons

Caspase-12, which is specifically expressed in ERS-induced apoptosis except death receptor- or mitochondrial-induced apoptotic signals, is sensitive to ERS [29]. CHOP is a marker that is up-regulated when excessive ERS activates all three signal pathways and also results in apoptosis. Therefore, the expression levels of caspase-12 and CHOP can determine whether ERS is involved in OGD/R-induced apoptosis.

**Fig. 2:**
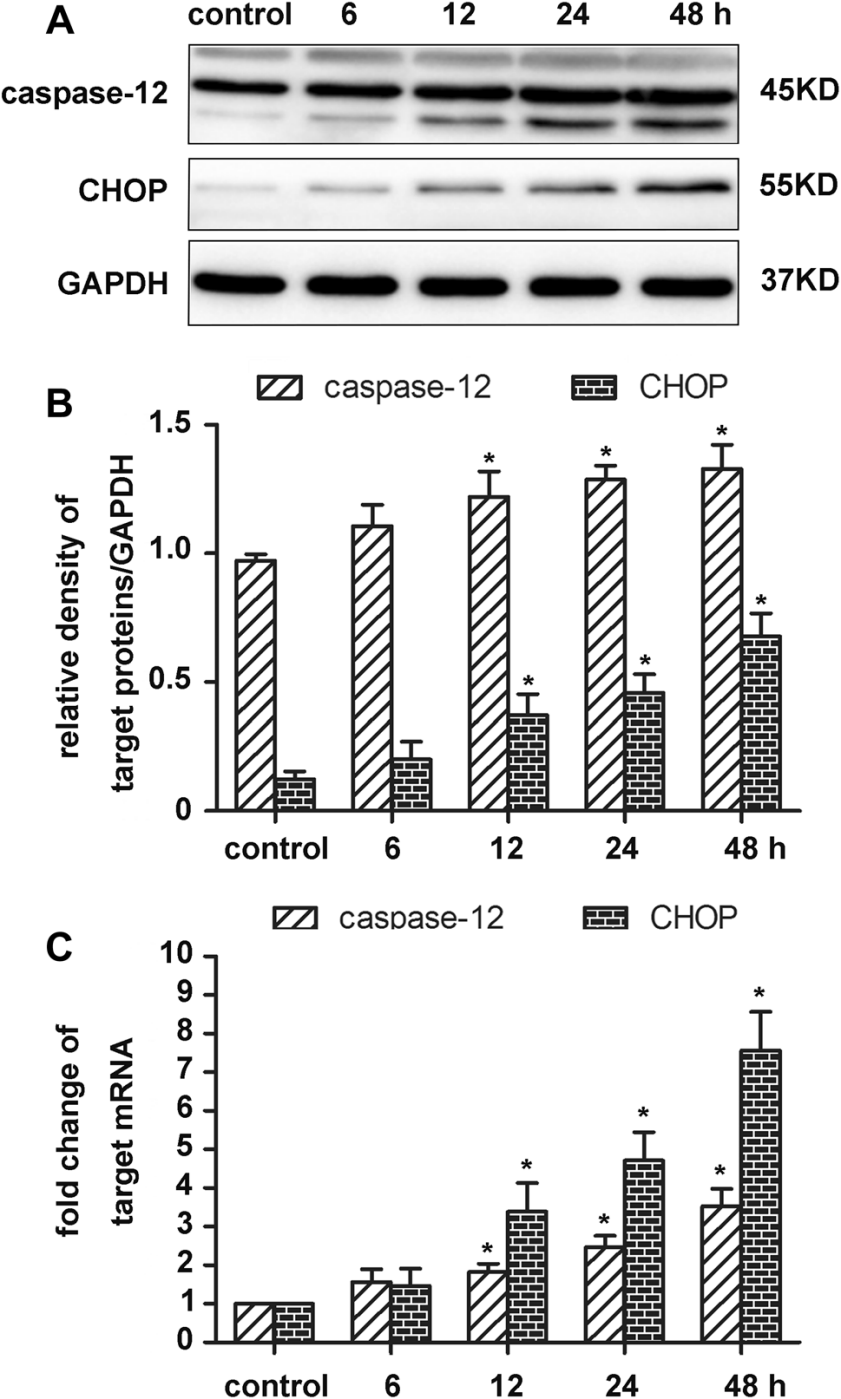
Effect of OGD/R on the expression of caspase-12 and CHOP in primary cultured neurons. **a, b** Protein expression of caspase-12 and CHOP in the OGD/R group. GAPDH was used for loading control and band-density normalization. Results represent the mean ± SD. *p < 0.05 versus the control group. The experiment was repeated six times. **c** mRNA levels of caspase-12 and CHOP in the OGD/R group. Results represent the mean ± SD. *p < 0.05 versus the control group. The experiment was repeated three times

Western blotting results revealed that the protein levels of caspase-12 and CHOP started to increase at 12 h and peaked at 48 h (*p* < 0.05; Fig. 2a, b). In accordance with Western blotting result, qRT-PCR results showed that caspase-12 and CHOP mRNA expression increased in a time-dependent manner from 12 to 48 h, peaking at 48 h (*p* < 0.05; Fig. 2c).

### Neuritin Decreased the Expression of GRP78, Caspase-12, and CHOP in Cultured Neurons Suffering from OGD/R

**Fig. 3:**
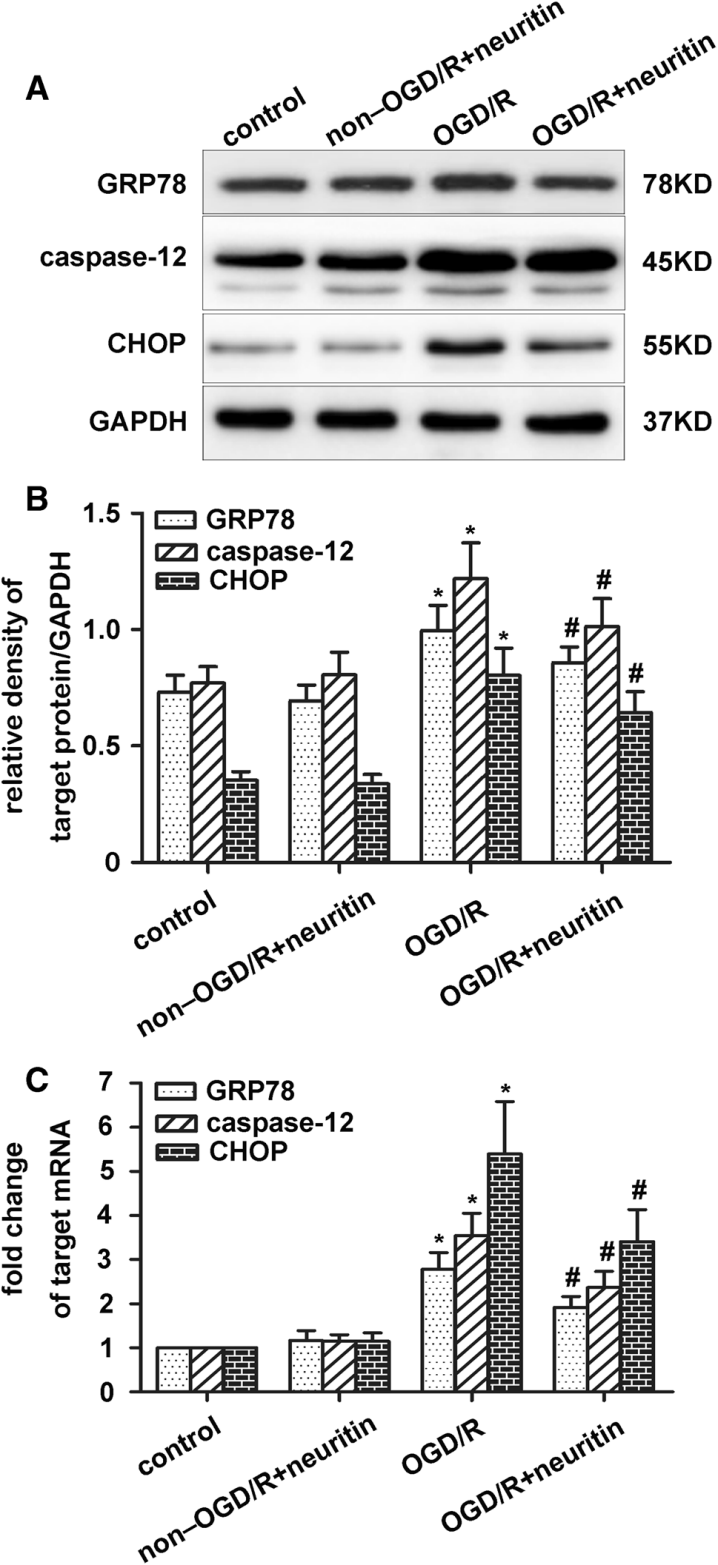
GRP78, caspase-12, and CHOP are decreased after neuritin treatment in primary cultured neurons suffering from OGD/R. After 24 h of OGD/R, the protein and mRNA expression levels of GRP78, caspase-12, and CHOP were detected by western blotting and qRT-PCR assay. **a, b** Protein expression of GRP78, caspase-12, and CHOP expression. GAPDH was used for loading control and band-density normalization. *p < 0.05 versus the control and non-OGD/R + neuritin groups, and ^#^*p* < 0.05 versus the OGD/R group. Data are the mean ± SD. The experiment was repeated six times. **c** mRNA expression of GRP78, caspase-12, and CHOP expression. *p < 0.05 versus the control and non-OGD/R + neuritin groups, and ^#^*p* < 0.05 versus the OGD/R group. Data are the mean ± SD. The experiment was repeated three times

To explore the effect of neuritin on anti-apoptosis in ERS, the neuritin-treatment group was dosed with neuritin (200 ng/mL) after OGD/R for 24 h. As was shown in Fig. 3, the expression of GRP78, caspase-12 and CHOP significantly increased in the OGD/R group (*p* < 0.05). Neuritin treatment significantly decreased GRP78, caspase-12 and CHOP protein expression levels compared with those in the OGD/R group (*p* < 0.05; Fig. 3a, b). The same results were found in the qRT-PCR analysis of GRP78, caspase-12 and CHOP mRNA expression (*p* < 0.05, Fig. 3c). No significant differenence was observed between the control group and the non-OGD/R + neuritin group (*p* > 0.05; Fig. 3).

### Neurons Apoptosis Rate was Ameliorated by Neuritin Treatment

**Fig. 4:**
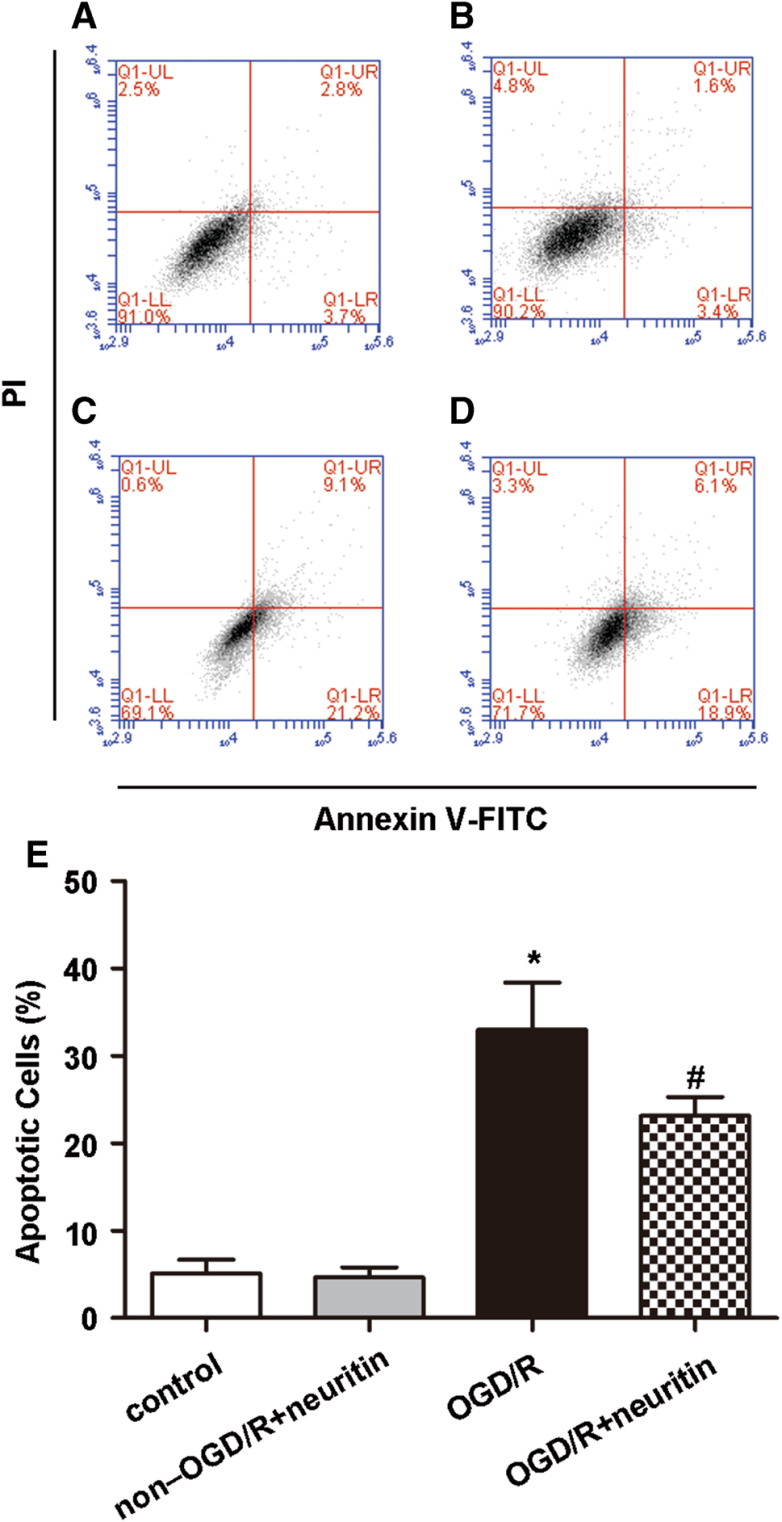
OGD/R-induced apoptosis was attenuated by neuritin treatment. After 24 h of reperfusion, the apoptosis of neurons was detected by flow cytometry. The figure shows a representative set of the dot-plot graph of flow cytometry analysis of the following groups: **a** control group, **b** non-OGD/R + neuritin group, **c** OGD/R group, **d** OGD/R + neuritin group, and **e** statistical result of apoptosis rate of neurons in different groups. *p < 0.05 versus the control group and non-OGD/R + neuritin group, and ^#^*p* < 0.05 versus the OGD/R group. Data are the mean ± SD. The experiment was repeated three times

Apoptosis plays a crucial role in the treatment and prognosis of neurologic disorders. In this study, we determined if apoptosis was altered by neuritin treatment by examining Annexin V and PI staining. As shown in Fig. 4, the cell-apoptosis rate including early (Annexin V-FITC^+^/PI^−^) and late (Annexin V-FITC^+^/PI^+^) periods of apoptotic process, increased in the OGD/R group (*p* < 0.05) compared with the control and non-OGD/R + neuritin groups. Furthermore, apoptosis rate decreased significantly after neuritin treatment compared with the OGD/R group (*p* < 0.05). No significant difference was observed between the control group and non-OGD/R + neuritin group (*p* > 0.05, Fig. 4).

### Neuritin Improved the Ultrastructure of ER

**Fig. 5:**
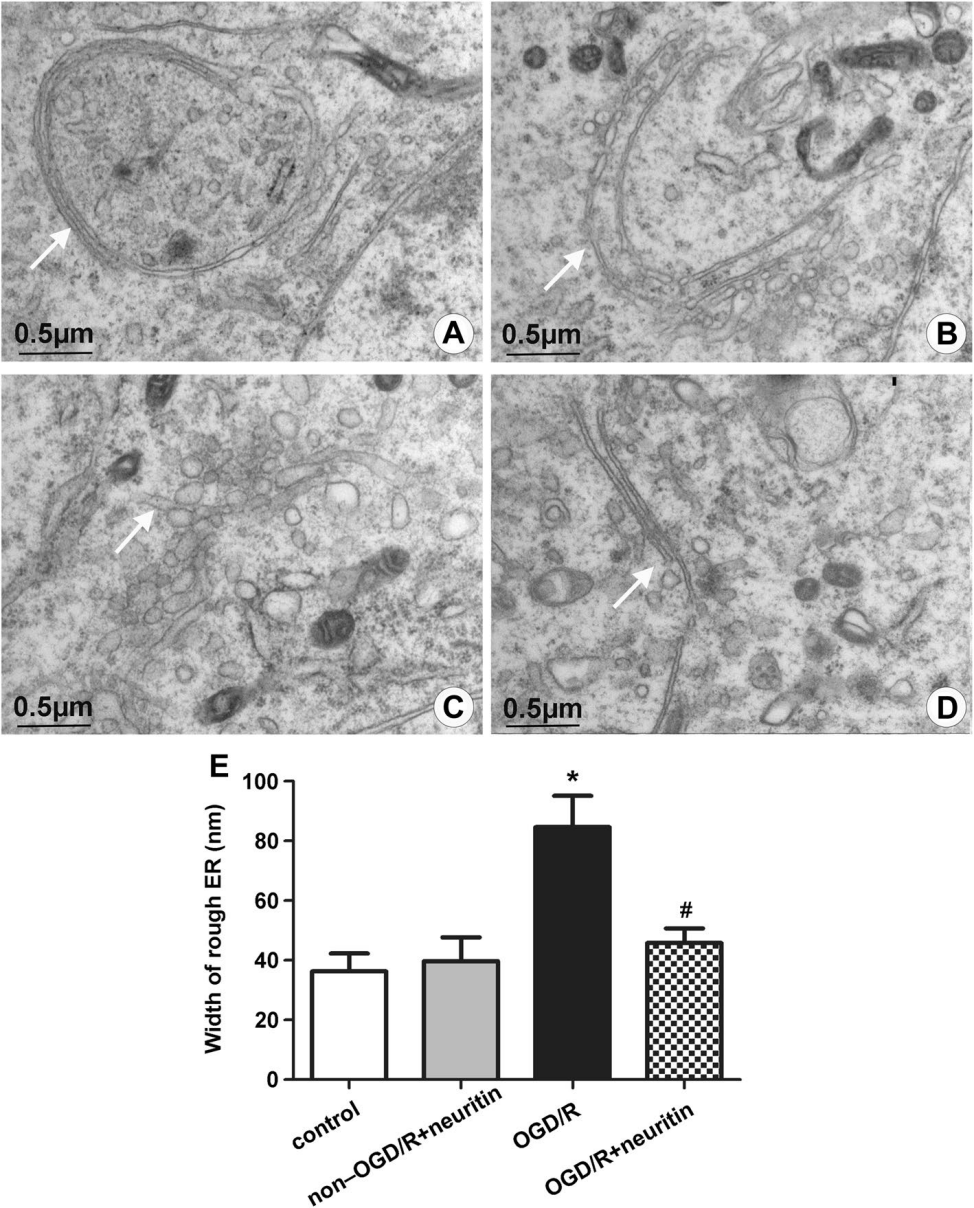
Ultrastructures changes of the rough ER of different groups were observed under a transmission electron microscope. **a** Control group, **b** non-OGD/R + neuritin group, **c** OGD/R group, and **d** OGD/R + neuritin group. Scale bar = 0.5 µm. **e** Statistical result of rough ER width in different groups. *p < 0.05 versus the control and non-OGD/R + neuritin groups, and ^#^*p* < 0.05 versus the OGD/R group. Data are the mean ± SD. The experiment was repeated three times

Newly synthesized proteins are assembled and modified within the cisternae of the ER, and the unfolded/misfolded proteins are concentrated mainly on ER [30]. Electron microscopy showed that the ER of neurons were rich and had narrow chambers and slender structures in the control and non-OGD/R + neuritin groups (Fig. 5a, b). After OGD/R for 24 h, the cisternae of ER expanded obviously, exhibiting different sizes of vesicles (Fig. 5c). In the OGD/R + neuritin group, the ultrastructure of rough ER improved when edema was relieved and when the size of cisternae decreased (Fig. 5d). This finding suggested that OGD/R induced apoptosis and ultrastructure changes of ER. Nevertheless, the neuroprotection ability of neuritin may contribute to the recovery of the ER ultrastructure.

## Discussion

In this study, the anti-apoptosis effect of neuritin was examined on an OGD/R model of primary cultured cortex neurons. We verified that oxygen and glucose deprivation is one of the inducements of ERS [6, 14]. Furthermore, neuritin treatment decreased the expression of GRP78 which is the initial factor of ERS. Additionally, apoptosis rate was alleviated, with decreased expression of caspase-12 and CHOP in the OGD/R + neuritin group after 24 h of reperfusion as dected by flow cytometry. The ER ultrastructure was also improved compared with the OGD/R group.

Our previous study has shown that neuritin exerts a neuroprotective effect on SAH in vivo and that neuritin attenuates the EBI after SAH by improving the clinical scale of brain edema and lowering neuronal apoptosis [11]. However, there are some inevitable factors in animal experiments, which renders the exact mechanism of these modifications unclear. In this study, we explore the possible mechanism of neuritin by using primary cultured cortical neurons in vitro. Furthermore, in a preliminary experiment, we found that the purified recombinant neuritin protein which was diluted with growth medium to 200 ng/mL had a better neuroprotection effect on primary cultured cortical neurons.

The apoptosis of neuronal cells is considered as one of the key factor after SAH and seriously effects neurobehavioral outcomes [12, 31, 32]. In this study, apoptosis was detected by flow cytometry after Annexin V-FITC/PI staining, and the apoptosis rate of neurons in the OGD/R + neuritin group was found to significantly decrease compared with that in the OGD/R group. Therefore, this finding suggested that neuritin treatment can reduce the apoptosis of rat cortical neurons after suffering from OGD/R, thereby, providing a new confirmation for the previous studies [11, 22].

GRP78 plays an important role in ERS and UPR. GRP78 acts as the initial molecular chaperone of the ER signaling pathway and is involved in translocating nascent polypeptides, facilitating de novo protein folding and assembly, targeting misfolded proteins to ER-associated protein degradation machinery, and maintaining calcium homeostasis [13, 16, 33, 34]. GRP78 also binds to three ER-localized transmembrane proteins of UPR containing IRE1, ATF-6, and PERK [5, 13] and these dipolymers maintain an inactive form under normal conditions [4, 35, 36]. However, under pathological conditions, GRP78 separates from the above-mentioned three proteins to release and activate these trasmembrane proteins, and UPR is initiated subsequently [33]. Moreover, excessive ERS damages ER physiological functions and exceed the repair-effect threshold of UPR in correct protein folding and processing [14] which results in the activation of the pro-apoptosis molecules of ERS downstream signaling, such as caspase-12 and CHOP [1, 12].

GRP78 overexpression is the beginning of UPR and is one of the most important self-protective mechanisms of cells. The levels of free GRP78 were monitored in the ER, and as a feedback mechanism, UPR was induced to restore GRP78 levels during ERS. First, GRP78 overexpression weakened UPR signaling. Second, UPR was activated after the GRP78 concentration decreased in the ER. Lastly, UPR weakened and GRP78 returned to the normal level because misfolded/unfolded proteins decreased [15]. However, cells were induced to apoptosis after suffering from a strong stress that was beyond the protection mechanism of UPR. Caspase-12 and CHOP, as the apoptosis-related factors, are involved in ERS pathways. Previous studies have indicated that caspase-12 can activate caspase-3 to trigger apoptosis, whereas CHOP can directly trigger apoptosis [37, 38]. Previous studies have indicated that GRP78 and caspase-12 increase after the OGD of cortical cultures [1]. Additionally, neuritin can reportedly to protect cultured cortical neurons from apoptosis by preventing caspase-3 activation [22]. However, the relationship between neuritin and ERS-induced apoptosis has been rarely reported. Therefore, on the basis of early animal experiment [11], an in vitro experiment was carried out to further explore the relationship. Results proved that GRP78, caspase-12, and CHOP significantly increased after OGD/R but decreased after neuritin treatment.

In summary, this study provided evidence of increased GRP78, caspase-12, and CHOP in primary cultured neurons suffering from OGD/R. Our findings suggested that neuritin played an important role in inhibiting neuronal apoptosis-induced by ERS. However, some aspects of our study deserve further exploration. The optimal concentration, administration time, and administration method of neuritin require further investigation. The effect of neuritin on the three pathways of ERS also warrants research attention. Therefore, more related studies will be conducted in the future.
